# Architecting Intelligent Smart Serious Games for Healthcare Applications: A Technical Perspective

**DOI:** 10.3390/s22030810

**Published:** 2022-01-21

**Authors:** Shabir Ahmad, Faisal Mehmood, Faheem Khan, Taeg Keun Whangbo

**Affiliations:** Department of I.T. Convergence Engineering, Gachon University, Sujeong-gu, Seongnam-si 461-701, Korea; shabir@gachon.ac.kr (S.A.); faisal89@gachon.ac.kr (F.M.); faheem@gachon.ac.kr (F.K.)

**Keywords:** internet of things, serious games, healthcare, task management, resource allocation

## Abstract

The immune system of human beings plays a pivotal role in guarding against different types of diseases. During the COVID-19 pandemic, people with weak immune systems were more likely to die. Regular physical activities and healthy food intake can significantly improve the immune system; however, people with a sedentary lifestyle and a busy job schedule find it challenging and tedious to maintain regularity. Different approaches have been used over the years to engage people in various physical activities and improve their mental and physical health. The concept of employing serious games (games whose primary purpose is not fun or entertainment, but a serious goal) to effectuate better results has become one of the popular choices among healthcare professionals and research communities. Internet of things (IoT) has enabled digital transformation with smart cities, smart infrastructure, and the fourth industrial revolution. There have been some relevant studies on the encouragement of serious games in healthcare in the past few years. However, few research studies encourage IoT-enabled serious games played with IoT devices (sensors and actuators) by making the game experience more ubiquitous and pervasive. Consequently, the adaptation of the IoT in serious games for healthcare applications is a massive gap despite its growing need in an era significantly affected by COVID-19. This paper discusses the possibilities of integrating serious games with IoT and discusses the standard architecture, core technologies, and possible challenges. Finally, we present a prototype architecture and its various components and a qualitative analysis with recent studies.

## 1. Introduction

Games have become an integral part of human life, not only for entertainment but also to help understand and learn serious jobs, such as education, strategic planning, and physical training. Serious games refer to the category of games in which the focus is tilted more towards strategies and learning rather than mere entertainment [1]. Serious games have proven to be remarkably productive not only as a learning tool in many domains, such as engineering, healthcare, physics, and history [2,3,4], but also in playfully achieving a particular job.

The gameplay in conventional serious games was based on underlying knowledge, which would often come from surveys and questionnaires [5] or based on their previous experience. However, such static data posed different challenges in terms of consistencies and accuracy, and thus future research encouraged the contextual data from sensing devices as a possible alternative. Internet of things (IoT) [6,7,8,9] is the realization of a vision where everything in the real world would interconnect and make smart decisions [10]. The plethora of miniaturized sensing devices generates real-time contextual information that can be exploited to make informed decisions [10,11]. Over the past few years, many application domains have embraced IoT to provide smart services and add more value to human lives [12].

Consequently, IoT has great potential to become a key enabler of modern-day gaming; a traditional game with dedicated terminal support can be converted to a smart game when played with remote mobile devices acting as an IoT node [13]. There are challenges for IoT connectivity of game agents, which are not yet among the standard approaches, and very few studies have indicated the potential use [14,15,16].

Nevertheless, serious games’ effectiveness has considerably increased with IoT node proliferation, sensing ambient scenarios, and providing real-time contextual information rather than human-based static data coming from questionnaires. Combining serious games with the IoT ecosystem’s interconnected physical hardware develops more data-driven games, better-informing games agents when the game progresses [14]. Consequently, there is a considerable rise in the use of IoT-enabled serious games. In some studies, such games are referred to as smart serious games (SSG) [13,17].

One of the challenges in SSG is the management of enormous volumes of real-time context data generated from sensing nodes. The introduction of Artificial Intelligence (AI) has revolutionized games and equipped them with powerful tools and procedures to make intelligent agents taking informed decisions. Modern-day games have become intelligent due to AI and machine-learning advancements. It is envisioned that Deep Reinforcement Learning (DRL) is poised to revolutionize games with a high level of visual understanding [18]. The success of DRL can be witnessed by AlphaGo, which is the first computer Go program to beat a three-year human champion. DRL received particular attention in gaming and redefined a new way to approach the game with intelligent background processes.

SSG is pervasive, and thus it demands an environment where users can play anywhere and anytime without the need to be in a specific premise. Ensuring fairness of play and synchronization of time and location is another challenge worth considering for successfully integrating serious games with the IoT. Similarly, SSGs can also pose security threats due to data transport and data persistence using public networks and clouds. Although edge and fog computing can overcome some of the limitations in using public platforms, they have limited processing capabilities and are yet to be investigated for computational intensive games.

Some studies [15,17] briefly considered integrating serious games with IoT and answered very primitive questions, such as the architecture change, game design change, and the communication protocol. Nonetheless, there is no concrete research on the architecture aspect of SSG based on an extensive literature study to present all the possible modules of SSG. Therefore, this article presents a technical perspective on designing SSG. The main contributions of this paper are as follows.

- To highlight the technical requirements of standardized architecture for SSG.
- To propose a core architecture, which can be regarded as a bare minimum with the optional add-ons.
- To highlight the challenges in realizing the standardized architecture.
- To present a prototype architecture and highlights its core processes.

The rest of the paper is organized as follows; Section 2 covers the background information and related works pertaining to serious games in healthcare. Section 3 presents the core design and model for a general SSG, while Section 4 presents the technical requirements and modules of the potential architecture. Section 5 presents the challenges in the realization of the architecture. Finally, Section 6 illustrates the prototype implementation of the potential architecture, and Section 7 argues the importance of the proposed architecture for recent studies. Finally, Section 8 concludes the paper and highlights different research directions.

## 2. Related Work

There have been quite a few studies aimed to encourage the use of gamification techniques [19] and some fun factors to accomplish a serious job. The related work of this article is summarized in three phases. The first phase is the pure serious games architecture without the use of contemporary machine learning technologies. The second one is intelligent serious games (ISG). The final phase, which is the primary goal of this article, is the SSG.

In the following subsections, the literature studies covering the architecture aspect of serious games across the above three phases are overviewed in the context of the proposed work, and the gaps in the current state of the art are highlighted.

### 2.1. Serious Games

Games played via a computer terminal have many facets. One of them is the ubiquity of gameplay. Prior to the integration of modern techniques, serious games were played using computers not only in the healthcare domain but also in education [20,21,22] and military training, to mention a few. The first reference to the term “serious game” was made back in 1970 to improve children’s intellectual abilities in the form of a board game. In 2002, important progress was made, and a unified forum was established—named a serious game initiative—whose central goal was to modify the existing form of video-based games to perform a serious job. The concept of serious games was backed in different early studies afterward.

The term serious game has been distinguished from gamification with distinct attributes. Gamification refers to the involvement of game elements in a generic application to encourage participation in a playful way. The rating system on Youtube, review scores on Publons, and ResearchGate profile are examples of gamification. There are some popular efforts in the form of serious games that have been made. The Oregon trial and Odyssey were developed to educate high-school children. Similarly, the Battlezone [23,24], Bradly trainer [25] were aimed for helping in training and strategies enhancement. There are other games, such as America Army, BiLAT [26] and Minecraft [27] to link the gaming industry with education [28,29].

### 2.2. Serious Games in Healthcare

Although the initial focus of serious games was the education domain and military, the recent past has witnessed an excessive amount of research in the gamification of healthcare services or deploying full-fledged games aimed to improve the health of human beings. Exergame [30,31,32] are a specialized category of serious games that aims to help players in performing physical activities through gameplay.

Dance Dance revolution and Wii Sports are popular games to encourage users to do healthy activities. Exergames can help in improving the metabolism, heart rate, and oxygen consumption, to name a few. Table 1 summarizes some of the studies that indicate a significant improvement in the health status of people using serious games or exergames.

### 2.3. Smart Serious Games

The involvement of machine-learning algorithms in healthcare industries has led to a new level. Patient history data, coupled with machine-learning algorithms, provide future insight into the status of the patients. However, the patient still needs to go to a hospital or medical facility to receive the service. Inspired by this problem, the internet of things technologies has enabled the patients to remain on their premises and have their status monitored from remote hospitals. In the same manner, SSGs are also a step towards realizing games designed for a serious job and must have the ability to be pervasive and ubiquitous.

The SSGs, in addition to the basic game requirements, have additional requirements imposed by the IoT. For instance, the resource management and allocation [39] on IoT nodes is challenging considering the seriousness of healthcare applications. In smart-healthcare-based serious games, various IoT nodes are sensors and actuators designed by different vendors; thus, the interoperability issue has to be addressed [40]. Similarly, the embedded IoT nodes have constrained capabilities; thus, security protocols should be optimized to meet the requirement of these miniatured devices [41].

Furthermore, there are other relevant challenges, such as the use of communication protocols [42,43], scalability [44,45], and analytical support of the massive context data generated using sensing devices [10].

### 2.4. Limitations and Research Gaps

There are still some of challenging areas that are yet to be addressed in the current state of the art. Despite the fact that serious games have proven to be effective in promoting exercise and physical activities [46,47,48], some of the limitations in these studies are the controlled environments because of the massive gap between elderly people and modern technology. Consequently, the induction of pilot trials are massively different than real-time deployments [15].

Another limitation is the scarce resources on the use of serious games and IoT in healthcare. There have been very few studies published in the recent past that suggested that this idea would enhance personalized engines and receive feedback from the environment. There are certain limitations that are discussed in the integration of serious games and IoT for other domains, such as education [17]. The architecture suggested by Henry et al. [14] suggested an improvement of student engagement to an extent but still lacked validation.

Moreover, the use of Node-RED and other third-party APIs suggest that the framework would not be a suitable choice for healthcare in which response time is a crucial factor. Deploying these APIs generally lower the response time; therefore, an alternative set of design patterns remains challenge for a healthcare scenario. The limitations of the existing approaches can be addressed with an architecture that can be easily used by the elderly citizens and people with no technical expertise. Moreover, due to the healthcare scenario, the focus of the proposed architecture is to address these challenges and provide a technical perspective on what can be the ideal starting point for the standardization of it in a conventional healthcare-based serious game.

### 2.5. Contribution

There are various studies on serious games architecture in the conventional paradigm, and there are many unstructured studies to suggest the architecture in pieces and parts. However, a standardized architecture is still a massive gap that integrates IoT requirements with serious games to make ubiquitous SSGs. To the best of the authors’ knowledge, this paper is the first attempt towards creating SSG.

## 3. System Model

**Figure 1:**
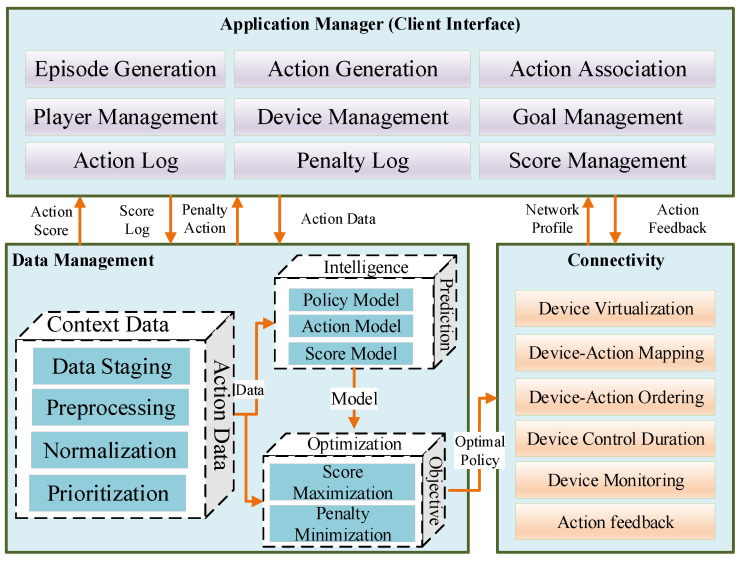
Conceptual architecture of an IoT-enabled serious game.

The proposed architecture aimed to have three major pillars; application management, data management, and device connectivity, as shown in Figure 1. The detail of each respective pillar is described in the following subsections.

### 3.1. Application Management

Architecture is the cornerstone of any application. The primary component of the architecture is the mechanism to interface with the outer world and the effective persistence of various components. The first pillar of the proposed architecture for SSGs in healthcare is the Application management and Client interface module. The Application Management module has four sub-components. The game composition forms the first component, which consumes game requirements and player-specific goals to auto-generate game episodes and their respective goals and winning criteria using natural language processing techniques (NLP).

The second component, the player management module, keeps track of player records, player registration, and player elimination in case of a penalty. The players can request using RESTful API to request for game participation. On successful approval, the player is added to the player log. In addition, the player can access specific IoT devices during gameplay. The device management sub-module should have a registry module for the auto-registration of IoT devices in the network. Another goal of the device management sub-module is to virtualize devices to make them addressable and discoverable with an Open Connectivity Foundation (OCF)-compliant URI, such as /*deviceid*, /*deviceprofile*.

**Figure 2:**
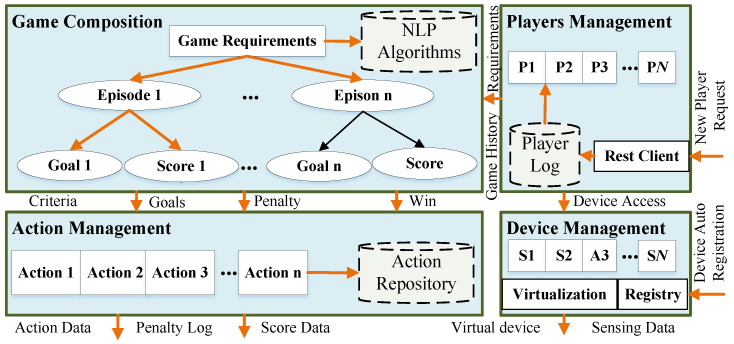
Modular representation of the application management component.

The final component is action management, where the primary aim is to generate actions based on the game episodes, winning criteria, goals, and penalties. It takes these parameters from the game composition module. These four layers expose action data, penalty log, device sensing data, and the player score and penalty history to the data management, which is the second pillar of the architecture. Figure 2 illustrates the modular representation of the application management pillar.

The Data Management module’s goal is to stage and analyze the contextual data generated from players’ devices and playing history to perform optimal decisions in future gameplay. The analysis is performed using a variety of algorithms, such as Deep Learning, Reinforcement Learning, and Optimization mechanisms to facilitate game users in taking informed actions based on their previous history and real-time context data generated from IoT Devices. The Data management module is considered the heart and mind of IoT-enabled Serious Games. At its core, it has various responsibilities.

First, the data coming from different game devices are raw data that are unusable for processing. Therefore, one role of the data management module is to preprocess the data and normalize them to a uniform format. Secondly, developing data collection mechanisms from health devices, such as pulse oximeters and ECG, and providing these to the game AI module is another crucial process inside the data management module.

**Figure 3:**
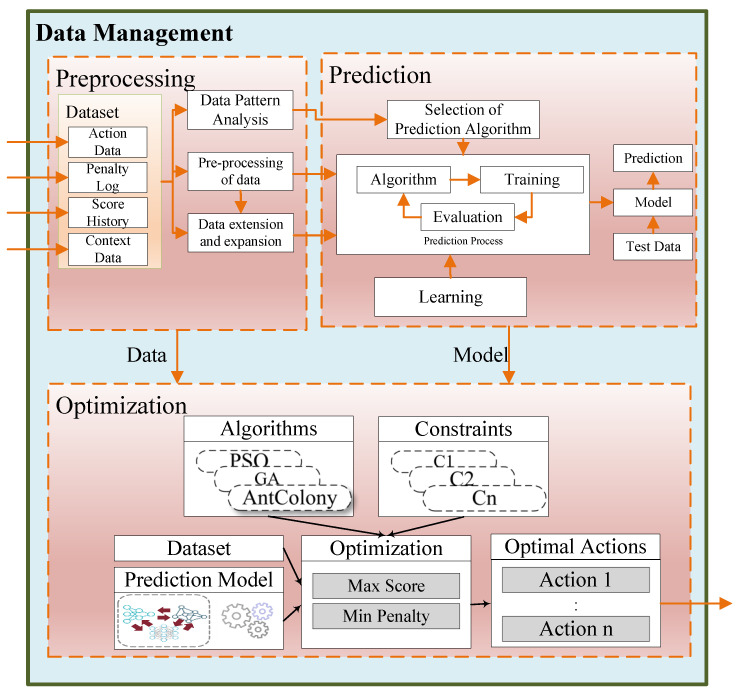
Block representation of the data management component.

Additionally, the game AI sub-component is responsible for preprocessing, prediction learning, optimization and investigating the constraint and different algorithms that can work better for a particular context and player. Moreover, it can employ deep learning (DL) and deep reinforcement learning (DRL) to iteratively consume the data and action and model the score and penalty of player actions under a given context. Furthermore, the model is provided to the optimization module, which optimizes the policy under given constraints (age and medical history) using particle swarm optimization, genetic algorithms, and mathematical solvers, as shown in Figure 3.

### 3.2. Connectivity

The connectivity module aims to connect the game actions (as part of the optimal policy) with the IoT devices and communicate on the physical layer according to the game requirements. It also investigates various popular wide-range communication protocols, such as LoRa, Sigfox, and Weightless to enable low-power long-range wireless communication among IoT devices to ensure IoT’s availability requirement with minimal power. The connectivity is based on a top-down methodology for connecting optimal actions with health sensors and actuators.

**Figure 4:**
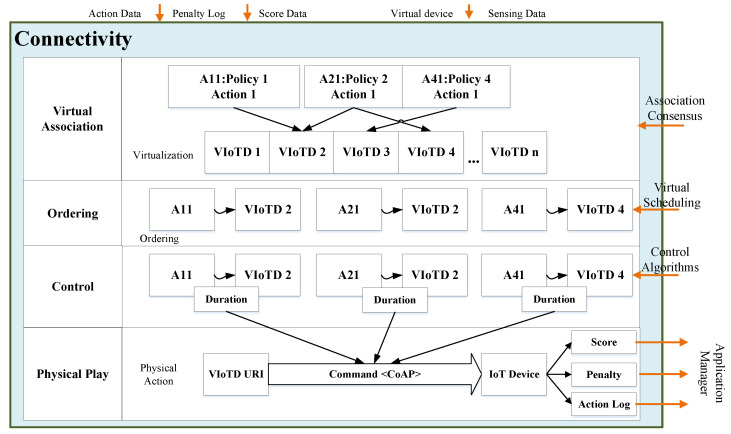
Flowchart of the IoT connectivity module.

Furthermore, to ensure a fair allocation with virtual scheduling and control duration using control algorithms is also one of the responsibilities of this component. The end goal is to perform the gameplay with health sensors and develop an algorithm to record each player’s score, penalty, and action log. Moreover, the player’s health assessment with parameters, such as game duration and action data provided to the application manager iteratively to be used in the future decision also constitutes a goal of the architecture, as shown in Figure 4.

## 4. Design Considerations

Architecture is the cornerstone for any application [49], and thus the critical issues for designing a sound architecture need to be elaborated. In this section, fundamental approaches and design considerations for designing serious games are investigated.

### 4.1. Modularization

The most tedious job in defining an architecture is its ability to be maintained with ease for future upgrades. A modular system conceals relevant processes in a single place, which makes it more robust due to high localization for errors. Similarly, the high modularity is achieved by relying little on other modules, also known as a coupling in the literature. One of the fundamental guidelines in software engineering practices is high cohesion and low coupling.

The proposed architecture is designed to be modular with a degree of cohesion that is high. The functionality of each module is defined concisely within the module. For example, the Captcha module is used to prevent bots from accessing the system. Similarly, OAuth2 is used to authorize the users. The key modules of the system are summarized in Table 2.

### 4.2. Decoupling

One of the most crucial aspects of SSGs in the decoupled nature of the modules. Decoupling is the process in which virtual players are independent of any IoT platform or service. These objects are not dependent on any application and can be consumed by any application. The primary motivation behind this work is decoupling and is considered one of the preliminary requirements of the system. Different IoT systems provide full-fledged services, including software, hardware, and user guidelines. Decoupling helps the target audience to customize the applications to meet the requirements of the domain. Furthermore, it allows the players to use virtual players as interfacing means for game service provision.

### 4.3. Web Services

A serious game played in the IoT space consists of different heterogeneous hardware. The interactivity among these devices has to be uniform despite the underlying heterogeneity. Furthermore, data from these devices must be exposed to administrative panels if the system is designed to be used without a user interface. Therefore, the proposed system is specifically designed to consume and provide data using restful web services. It is clear that RESTful services are the de facto standard for IoT platforms. Thus, vital design considerations for the architecture include its ability to support RESTful web 196 services and provide secure end-to-end communication between different modules in the SSGs platform.

### 4.4. Authentication and Role Management

Roles and permission play essential roles in the usage of any system. Without proper role management and permission handling, a player can see the progress or the penalty log of irrelevant data or even, in the worst case, can access the administrative panel to modify the game data. This will make gameplay extremely unfair. Therefore, authentication and role management are vital design consideration features that allow users to associate with specific roles, such as administrative roles.

This provides the user with the privileges to read and write data while using the proposed system. For example, the player associated with its virtual representation can only read data, whereas the virtual player provider can read and write data simultaneously. However, all users are authenticated before using the system. This allows the system to be secure, and anonymous users cannot access its resources

### 4.5. Authorization

Authentication and authorization are two different modules. Authentication is required only for identifying the user, whereas authorization is needed to check whether certain actions can be performed by a specific user or not. For example, even if the user is authenticated before using the system, the user can only read the data and cannot delete the virtual object. Although there are different authorization methods, we used the OAuth server to provide security in our proposed system.

### 4.6. Security

IoT applications use different low-powered devices that share and receive data over the internet. The low-powered devices have constrained capabilities, and full-fledged security algorithms can drain their power. Therefore, the security of IoT applications has to be given different consideration compared with conventional applications. Without security, the system is incomplete. System security is considered one of the most fundamental parts of any system. If the system is not secured enough, it can be exposed to hackers. The proposed system is deployed on the cloud to provide an extra layer of security. Open-source modules, such as Secure login and Captcha, can be added to the core to make the system more secure.

### 4.7. Data Analytics

Context-awareness and user preferences are fundamental parts of any application architecture. The purpose of context-awareness is to display the relevant data to the users. For instance, a user in the automobile industry is only interested in resources integrated into auto-mobile scenarios. Similarly, a user belonging to a smart factory is only interested in content related to the smart factory. The proposed system is designed to learn user preferences and is intelligent using an analytics module, which can further help during gameplay.

## 5. Prototype Implementation

Based on the guidelines and model that we presented in earlier sections, a prototype was implemented that reflected the simplest architecture of the game management dashboard and underlying model. As part of the implementation, Python programming language was used as a back-end language to store and fetch the data from the sensors associated with the different players. The sensors can be attached to the players using a wireless body area network or built-in sensors via a smartphone.

For simplicity, the smartphone approach was followed. The implementation stack is depicted in Table 3. Python was used as a core programming language to develop the application interface. The data generated from different players’ smartphones was supplied to the application module for data analysis. For this, we used Samsung Galaxy Fold 3 phones with different health-related applications. Data analysis and machine learning were implemented in Pandas and TensorFlow libraries of Python.

Furthermore, we deploy popular algorithms using Scikit Learn (SKlearn) library. This library has built-in modules for State-of-the-art classification and prediction modules—Decision tree, Random Forest, and Logistic Regression, to name a few. For visual appearance, Bootstrap 3 and Javascript are used. Google MAP API is used for the real-time location of different players in the dashboard. There are two spaces: the cyber plane and physical plane.

**Figure 5:**
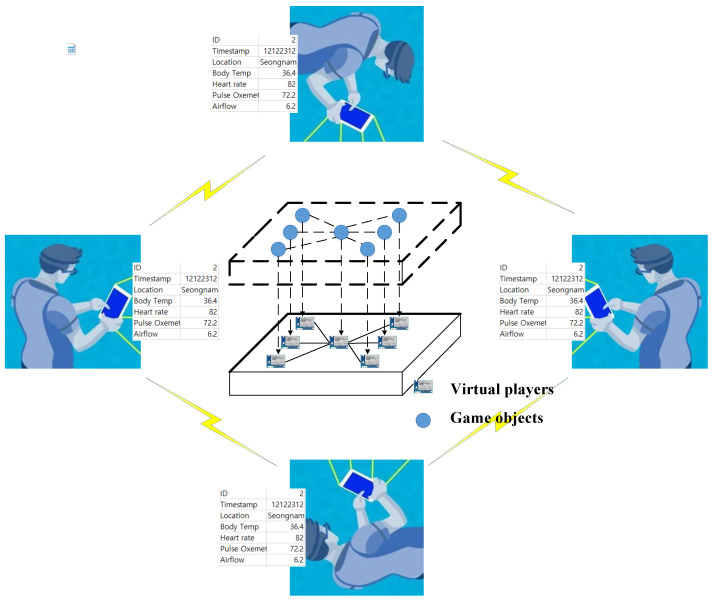
The flow of data between physical space and cyberspace.

Conceptually, the players, along with their smartphones or smartwatches, form a physical space. The connectivity of players using smartphones and data transfer using application-level protocol makes it a typical IoT application. The client application running on players’ devices generates data services that are consumed by an application platform. The platform is hosted in cyberspace, which consumes the data with the client. Different operations in the prototype are shown in Figure 5.

Different players from around the world transfer the data using smartphones to the nearest servers. The players can be from around the world. The data can be player ID, location, heartbeat rate, airflow rate, and pulse oximeter, to name a few. Every player has a virtual representation in cyberspace, also known as a virtual player. The virtual player attached with the game object in cyberspace monitors the data during the gameplay. The data is pushed to the main application server, which processes the data and forecasts player action and scores. The application server runs an administration module that monitors the location and gameplay of different players in real-time. The main modules of the prototype are given below.

### 5.1. Administrative Interface

**Figure 6:**
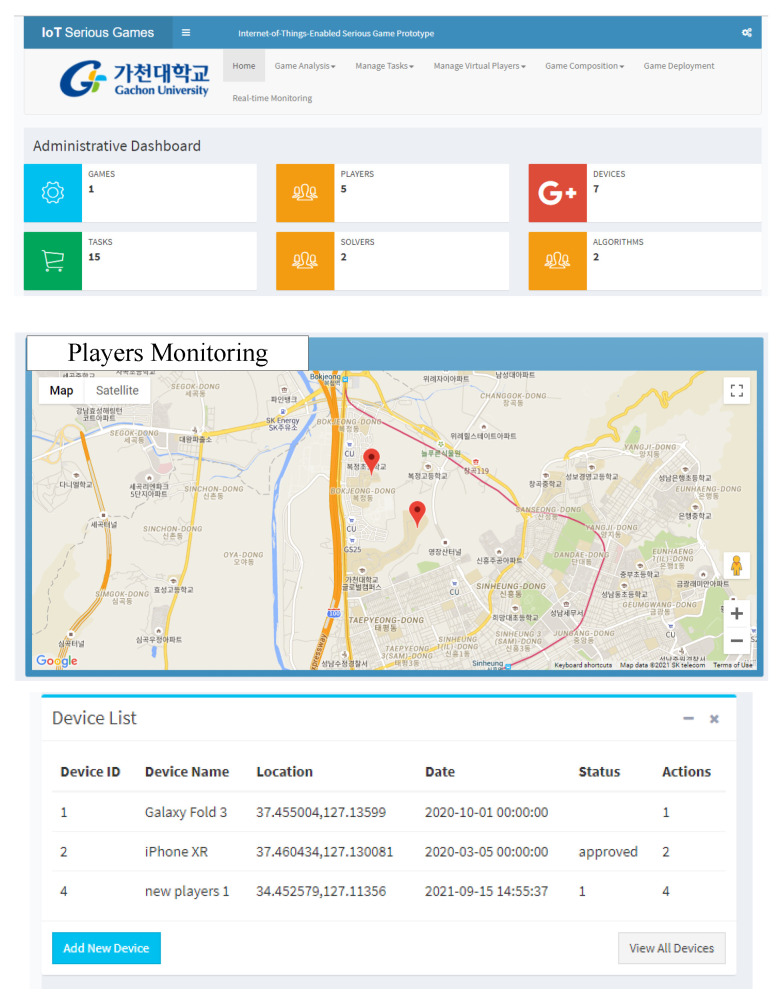
Administrative interface of the proposed architecture.

The main page of the prototype application is a comprehensive space for monitoring different players’ activities in the game process. There are different navigation items for various processes. Beneath it is the game’s statistics concerning the number of active players, number of tasks, number of devices, number of algorithms, to name a few. The location of the player is monitored in real-time using Google MAP. There are different listings, such as a device list, player list, activity list to form an administrative space where these game objects are monitored, modified, and removed upon the game termination. Figure 6 shows the main dashboard of the prototype application.

### 5.2. Game Management

The management of different players and game activities is performed with various state-of-the-art processes. First, the players’ activities and games are predicted based on the user profile and historical data. Similarly, the cost and penalty of a particular step are computed using optimization methods. Thus, we must name the underlying algorithm to assess the game activities using the data as a solver. Thus, a solver can have numerous types and roles. For instance, an optimization solver finds an activity with the maximum cost and least penalty among possible activities. Similarly, prediction solvers provide a model trained on the data to predict the cost and penalty of a particular event. There are other solvers as well that solve other issues, such as scheduling activities using different scheduling algorithms.

Various processes from the inception of game requests to the deployment of gameplay are summarized in Table 4.

**Figure 7:**
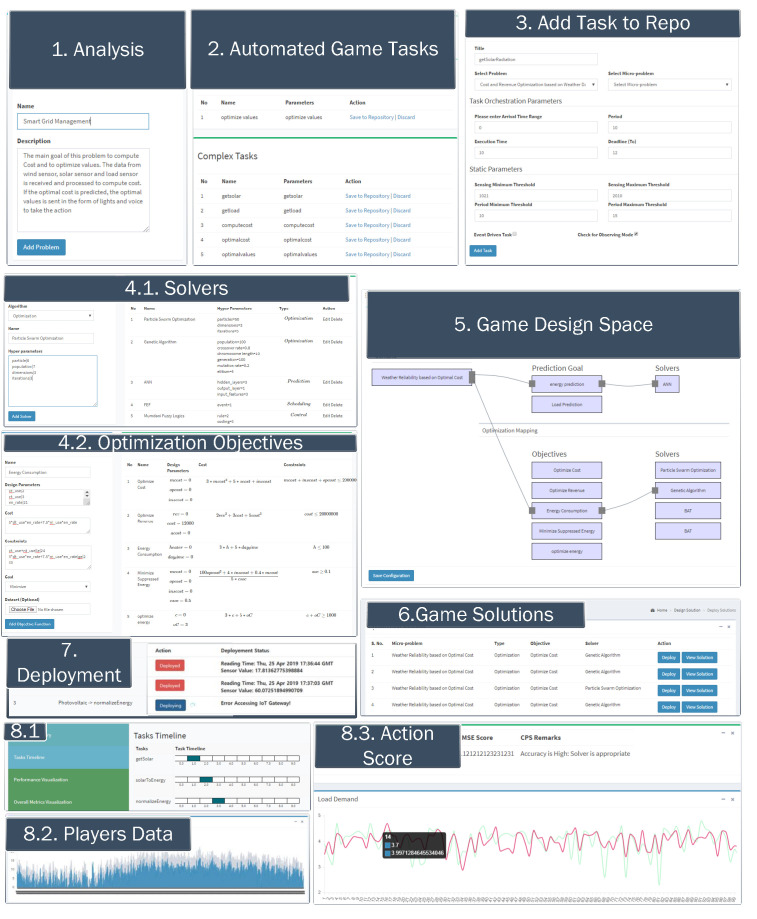
Administrative interface of the proposed architecture.

The overall process concerning the administrative perspective is portrayed in Figure 7. The screenshots of each interface highlighted in Table 4 are portrayed as sub-processes as shown in Figure 7.

### 5.3. Game Data Management

One of the crucial aspects of the prototype is the ability to interact with the physical players and the sensors each respective player carries. Players can register their device using REST APIs. The type of the data and the call parameters are explicitly stated, and if a valid request is made, a connection of device with the architecture is established as a result. In the initial stage, the data taken from different sensors associated with players and the environment in which the game is played are recorded and stored. Table 5 summarizes the simplest deployment model and the context data types.

## 6. Challenges

Recently, extensive research has been conducted in serious games based on AI and IoT. This section describes the challenges involved during the design and development of serious games. The overview of the challenges involved in designing serious games and their respective future directions is given below.

### 6.1. Conceptual Architecture

SSG is a relatively new paradigm, and, currently, much work is being carried out. Conceptual architecture is a fundamental part of any platform. Different methodology and techniques have been proposed to integrate AI and IoT in serious games to improve its architecture and enhance its capabilities for the healthcare domain. Researchers are attempting to integrate serious games with IoT and ubiquitous computing. The proposed framework does not reflect the full potential of SSGs. Current studies lack a fundamental part of the architecture, and hence there is a massive gap in the current state of the art.

The conceptual architecture should fill the gap between the real-world space and the gaming industry by satisfying the requirements of both IoT and serious games. IoT devices form a heterogeneous network, and thus there must be a module in the conceptual architecture that deals with the IoT device registry and access irrespective of their vendors. Existing architecture needs to be investigated appropriately and upgraded to meet the industry standards.

### 6.2. Smart User Interface

The usability of any application is one of the essential aspects to determine if it can accomplish specific goals in an efficient, satisfactory, and effective manner. Therefore, usability tests are conducted to determine the feasibility of a system. Recent advancements in intangible computing have led to increased interest in developing smart user interfaces. Still, there are open spaces and challenges to imply machine learning techniques to more intelligent interfaces. User interfaces commonly involve human-to-computer interaction, but currently, research is being performed for brain-to-computer interactions such that the user interface is more intelligent and intuitive.

### 6.3. Scalability

Scalability is one of the significant concerns regarding IoT-enabled platforms, as there is a massive increase in the number of devices and users involved in a particular game. As the number of users and resources grows, scalability issues also increase. There are three dimensions of scalability, i.e., administrative scalability, size scalability, and geographical scalability. The architecture of serious games plays a vital role in handling the number of devices and users. However, there has been little research done in the gaming industry. One approach to resolve the scalability issue is to deploy edge nodes. However, node edges can arise many other challenges, such as reliability, robustness, and security.

### 6.4. Auto Adaptibility

Technology is moving towards an autonomous environment. With the use of artificial intelligence, tasks performed by humans are being replaced by machines. It clear that machines can already outperform human beings in many tasks. For example, Go is a top-rated game for AI because of its complexity. AlphaGo is an AI program designed to play the Go game autonomously. As a result, it defeated the World champion human player. Autonomous cars are undergoing testing and will soon be available in the market. Autonomous agents can find the optimal solution for any task that human performs. For example, in a chess game, the autonomous bot can predict the next moves based on its previous data and perform its move accordingly. Even though much research is being conducted with AI, a more empirical study is needed to enhance the AI algorithms in the gaming industry.

### 6.5. Resource Management

The IoT is an internet-based platform for interconnected devices and applications. Since IoT devices have constrained abilities and massive network traffic, this leads to various issues, such as power issues, congestion on IoT devices, and devices being under-utilized. Consequently, it will lead to resource management issues. Recent studies suggest many approaches, such as resource-aware task scheduling to handle resource management issues. However, they solve the congestion issues but with a minimal dropping rate. However, more research is needed to improve the algorithms to use IoT devices fully in the gaming industry.

### 6.6. Virtualization

A virtual object is a digital representation of a real-world object capable of interpreting, communicating, and providing relevant services. Virtualization faces many challenges, such as interoperability, scalability, security, and privacy issues. Interoperability is one of the major issues in virtualization-based IoT solutions. The reason is that there are no specified standards that IoT devices with different architecture can communicate with each other. Another primary concern is scalability. Upsurge in IoT-based solutions will exacerbate the scalability issue in the future.

Due to scalability, security and privacy concerns also arise. The virtual objects need to be appropriately managed, i.e., they should be secured, updated frequently, and deleted as soon as the life cycle ends. Virtualization plays an intrinsic role in resource management. It has the potential to resolve the scalability issue by providing a platform that can easily manage diverse physical IoT nodes.

## 7. Architecture Evaluation and Discussion

The proposed architecture is evaluated using essential requirements of serious games and IoT and identifies different strategies for standardization of the architecture.

### 7.1. Quantitative Assessment

One of the crucial measures for the quantitative assessment of a novel architecture is to expose it to an unknown load and oversee its behavior [50,51]. For carrying out quantitative evaluation, load testing of the architecture is performed against well-known key performance indicators, such as the response time, throughput and latency for a varying number of simulated players. For this, Apache JMeter is used, which is an open-source tool to automate test scripts under different simulated environments [50]. The cross-origin API calls using REST services are generally slower than the local calls. Therefore, we tested the architecture in a scenario where many simulated players make an API call to access certain resource or sensing data. Table 6 provides a summary of the key performance measures for 300, 500, and 1000 simulated players.

There are various scenarios in which the proposed architecture can be assessed. As shown in Table 5, numerous resource endpoints can be accessed to obtain the context data. If the resource resides in a remote IoT server, a cross-origin request is made via an API call whereas, if the resource is already available in local data server, then no API call is made. In order to evaluate the worst response time on peak load, certain API calls are randomly made by simultaneous players and the response time is recorded.

**Figure 8:**
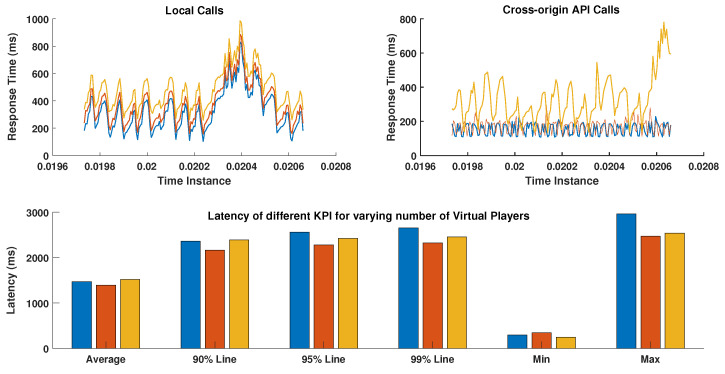
Quantitative analysis of resource endpoint access by simultaneous players.

As discussed earlier, 300, 500, and 1000 players are considered for a short time interval as shown in Figure 8. The response time for local calls increases proportionally with increasing simultaneous players. However, in the case of remote API calls, the response time is uniform until 500 players. For 100 players, the response time increases significantly and also portrays a random behavior. Nevertheless, even for 1000 players making simultaneous requests to external resource points, the response time is still in a range in which the system can be characterized as stable.

The bar graph represents numerous KPI provided by the Apache JMeter to evaluate the load testing with different scenarios. The maximum latency for 1000 users is touching 3000 ms. Moreover, these measures are effective in modeling the worst-case scenarios of any architecture, thus a threshold line can be defined after which all the requests are automatically timed out or re-initiated, thus, avoiding the indefinite wait for the resource.

### 7.2. Qualitative Assessment

There are specific architectures and design patterns for serious games. However, the integration of serious games with IoT brings new challenges. In addition to the requirements of conventional games, IoT-specific requirements, such as ubiquity, scalability, security should be addressed in a candidate architecture. Table 7 overviews state-of-the-art techniques and prototype to address a common issues of IoT-enabled serious games. For the evaluation purpose, we have highlighted nine crucial qualities for serious games played in the IoT environment.

These include ubiquity, security, data analysis, standardization, resource allocation, and prototype development. Standardization is considered a fundamental challenge as there have been many efforts recently done in a domain-specific way. Such studies solve a problem of a very narrow scope. However, these can be investigated to form a generic architecture to help researchers and technologists to start from an already-existing design pattern.

Moreover, most of these studies consider sensing devices and IoT gateways, but their study on resource allocation is shallow. In the same way, some of the design architecture is more biased towards a data-intensive approach and overlooks the challenges posed by the IoT. For instance, the studies in [52,53] tilt more towards a data-intensive approach and consider data analysis as the central approach towards the design of IoT-based serious architectures.

On the other hand, the work in [13] focuses more on network infrastructure and the proper deployment of IoT nodes while considering the data analytics as a secondary vital aspect in the design. It also discusses the resource allocation, but that is partially addressed and warrants further investigation. Thus, the studies under evaluation have shortcomings, especially in terms of designing a balanced architecture.

Nevertheless, some of the studies [14,54] are comparably more balanced and tackle different parameters outlined in Table 7. However, they are carried with a narrow scope in mind and thus cannot be generalized. The standardization of the architecture remains an issue worth investigating. Since the potential architecture should be generalized and balanced in addressing all the requirements of serious games and IoT, the prototype illustrated in this paper is an approach much closer to the goal than the state-of-the-art. The proposed architecture has data analytics, resource allocation, ubiquity, and standardization, to name a few. Thus, it can be considered a standardized architecture.

Nonetheless, the proposed architecture has certain limitations. First, the devices are very diverse in this phase, and thus the security protocol and communication protocol stack are not defined. Similarly, relevant to the mentioned limitations, interoperability also has a question mark considering the hardware is not known in advance. Second, data analytics is partially implemented on the cloud. However, in the healthcare scenario, the trained model may not reach the required response time, and thus other approaches, such as edge deployment are worth investigating in the future.

## 8. Conclusions

In this paper, we discussed the possibilities of serious game integration with IoT and AI technologies to develop an effective tool for improving the health of people with a sedentary life style in a post-COVID area. The SSG has a massive potential to improve the health of people in an interesting way. The tough work schedule and bad eating habbits among adults can make them lazy and at times they find regular physical activities tiresome. Such games can give them a sense of competition and at the same time improve their health in a more playful manner.

In this research, we highlighted the standard three-layered architecture that forms the stepping stone of the basic architecture. The integration of IoT and AI brings massive benefits, such as player history, score forecasting and other fun aspects in the game play. However, it brings some hurdles due to the distant placement of different players. We highlighted some of the design considerations and challenges that have to be considered for its realization.

We developed a simple administrative application prototype and discussed various interfaces and functional processes. In this paper, we presented a technical perspective and the possibilities of its realization with relevant references to existing state-of-the-art. In the future version of this research, we will investigate a full-fledged serious game with working machine learning models in a more real environment and carry out a survey on the effectiveness of such games.

### 8.1. Implication for Healthcare Industry

This architecture can be effectively utilized by healthcare therapists for general patients who want to improve their healthcare without conventional drugs. The proposed architecture can help them interact with similar-mind players from all around to globe to involve themselves in a fun activity that can also be effective towards their health. The previous case studies of different patients can be recorded in the data management part, which can be later on accessed by a health therapist to monitor any possible improvements. Additionally, this architecture can provide a unique dataset for analysis, which can be helpful to mine certain patterns of an individual who has interacted with the platform.

### 8.2. Implication for Academia

Apart from the practical implications in the healthcare industry, the proposed architecture can be utilized in academia as a learning tool. Medical students can analyze variations in the generated data under different circumstances and conditions. For instance, the data from airflow sensors taken while a person is stationary will be subtly different when taken from a moving person. Similarly, different variations of conditions and the impacts on health data can be analyzed and can provide an effective learning environment for medical students.

## Figures and Tables

**Table 1:** Summary of different studies supporting the use of serious games for healthcare.

Reference	Purpose	Results
Active Video Games [32]	Propose active video games with the Wii Fit to increase the energy expenditure and physical activity	Improved heart rate
Exergame using Playstation 2 [33]	Healthy male volunteers completed a group of games and their results were recorded	Energy Expenditure, Blood lactate (BLa), Fat and carbohydrate oxidation
Physically interactive video games [34]	19 college students completed different activities using Dance Dance Revolution game sessions	Heart rate, Perceived exertion, respiratory exchange rate, oxygen consumption
Interactive video/arcade game [35]	13 male and female students participated and familiarized with three games	Heart rate, Energy Expenditure, Caloric expenditure
Interactive video game with stationary cycling [36]	Combined game with stationary cycling compared to traditional aerobic training	VO2, Systolic blood pressure, vertical jump
Virtual reality game [37]	Virtual reality environment for cognitive training of daily life activities	neuropsychological rehabilitation, memory and attention functions
Fitness-themed video games [38]	Games played using motion controller can help in enhancing energy expenditure compared to traditional games	Energy expenditure

**Table 2:** Summary of different modules used in the prototype system.

Module	Description
axonomy	Taxonomy is used to categorize virtual objects.
MySQL Adapter	MySQL adapter is used for basic DB operations, such as select, create, edit, and delete.
Views	This module allows user to search for the relevant content on the platform.
HAL	Hypertext Application Language (HAL) supports RESTful web services that include embedded objects and hypermedia.
RESTful APIs	RESTful APIs are used for commands like POST, PUT, DELETE.
Authorization	OAuth2 provides security layer and is used for authentication of users.
Serialization	Serialization module is used to serialize JSON or XML request payload.
Captcha	Captcha is used to prevent anonymous users from accessing the system.
Secure Login	This module provides security in login form.

**Table 3:** Summary of the different modules used in the prototype system.

System Component	Description
Operating System	Android, Microsoft Windows 10
Hardware	Galaxy Fold 3, Galaxy Watch 4
Memory	8 GB
Server	Flask Webserver
Libraries	Jinja, HTTPClient, Bootstrap, Javascript, HTML 5 and CSS3, JSPlumb, RESTFul API
IDE	PyCharm, Sublime Text
Core Programming Language	Python 3.7
Backend Persistence	MySQL
Machine Learning Libraries	Pandas, TensorFlow, SKLearn

**Table 4:** Technology stack of the proposed architecture.

Technology	Description
Game Tasks Generation	The tasks are generated using the game requirement by employing different NLP solvers.
Solvers	Various solvers to map different state-of-the-art algorithms in a certain phase, e.g., prediction and optimization.
Objective Management	Objective function definition for increasing the game rewards and decreasing the penalty.
Game design space	The design space to assign different solvers to various game activities.
Deployment	The allocation of tasks on physical devices and the sensing data generation as a response.
Scheduling	Game activities scheduling.
Data visualization	The visualization of sensing data in real time during game play.
Solvers quality	Interface for assessing the quality of a certain algorithm.

**Table 5:** Overview of data access points from different devices connected to IoT gateways.

Sensor	Association	IoT Gateway	Communication Protocol	Data	Sampling Interval	API Endpoint	Response
Airflow	Player	Arduino	HTTP	Breathing data	Periodic, 5 s, Event: Abnormal	/get-airflow	JSON
Pulse Oxymeter	Player	Arduino	HTTP	Pulse, Oxygen Saturation	Periodic, 5 s	/getPO	JSON
ECG	Player	Arduino	HTTP	ECG Data	Periodic, 10 s, Event: Abnormal	/getECG	JSON
Temperature	Player	Raspberry PI	HTTP	Temperature Data	Periodic, 5 s, Event: Abnormal	/getTemp	JSON
BLE Beacon	Environment	Android, iOS	MQTT	User Location using RSSI	Publish-Subscribe	/getLoc	JSON
PM Sensor	Environment	Raspberry PI	MQTT	Particulate Matter (PM25, PM10)	Publish-Subscribe	/read-dust	JSON
Smart watch	Player	Android, iOS	MQTT	Steps data	Periodic: 2 h, Publish-Subscribe	/read-steps	JSON
CO_2_	Environment	Arduino	HTTP	CO_2_ data	Periodic: 10 s	/getCO_2_	JSON

**Table 6:** Overview of Data access points from different devices connected to IoT gateways.

Virtual Player	Samples	Average	Median	90% Line	95% Line	99% Line	Min	Max	Throuhghput	Received Data (kb)	Sent Data (kb)
300	300	110	117	131	138	159	71	213	3.006012024	4.632311498	0.469689379
500	500	111	115	133	141	176	69	352	5.002401153	5.315051225	0.766969708
1000	1000	1787	1969	2834	3048	3278	64	3861	9.884841595	5.000339791	1.544506499
TOTAL	1800	1042	741	2606	2869	3224	64	3861	5.981656254	4.982766391	0.929765905

**Table 7:** Qualitative evaluation of the recent work towards the integration of serious games in the IoT.

Architecure	Domain	Resource Allocation	Interoperability	Ubiquitity	Data Analytics	Security	Standardization	Game Prototype
Proposed	General-purpose	Yes	partial	Yes	partial	not yet	Yes	yes
Hai Huang et al. [54]	Cultural learning and education	no	no	yes	image data only	no	no	yes (simulator)
John Melthis et al. [13]	Topology of Serious Games	patial	no	yes	no	partial	yes (network only)	no
Hai Huang et al. [52]	Card-based IoT game	no	no	partial	yes	no	no	no
H. Engström et al. [53]	Serious games design knowledge	no	no	no	yes	no	yes	no
John Henry et al. [17]	Framework for Integrating Serious games with IoT	no	yes (Broker model)	yes	yes	no	no	yes
John Henry et al. [14]	Randomised Control Trial through serious games	no	yes (Broker model)	yes	yes	no	no	no
Jun Qi et al. [55]	Hybrid Hierarchical Framework	no	no	no	yes	no	no	yes

## Data Availability

Not applicable.
